# Assessing the Role of Climate Change in Malaria Transmission in Africa

**DOI:** 10.1155/2016/7104291

**Published:** 2016-03-15

**Authors:** E. T. Ngarakana-Gwasira, C. P. Bhunu, M. Masocha, E. Mashonjowa

**Affiliations:** ^1^Department of Mathematics, University of Zimbabwe, P.O. Box MP 167 Mount Pleasant, Harare, Zimbabwe; ^2^Department of Geography and Environmental Science, University of Zimbabwe, Harare, Zimbabwe; ^3^Department of Physics, University of Zimbabwe, Harare, Zimbabwe

## Abstract

The sensitivity of vector borne diseases like malaria to climate continues to raise considerable concern over the implications of climate change on future disease dynamics. The problem of malaria vectors shifting from their traditional locations to invade new zones is of important concern. A mathematical model incorporating rainfall and temperature is constructed to study the transmission dynamics of malaria. The reproduction number obtained is applied to gridded temperature and rainfall datasets for baseline climate and future climate with aid of GIS. As a result of climate change, malaria burden is likely to increase in the tropics, the highland regions, and East Africa and along the northern limit of falciparum malaria. Falciparum malaria will spread into the African highlands; however it is likely to die out at the southern limit of the disease.

## 1. Introduction

Climate change is projected to alter the distribution of vector borne diseases and malaria is no exception. Children under five years of age and pregnant women continue to be at risk. One of the key millennium development goals is to halve, halt, and reverse the scourge of malaria by 2015. The disease has not yet been halved, although a significant reduction in malaria incidences has been recorded [1]. These little gains achieved to date are under threat from climate change.

Malaria is sensitive to climate change in the sense that the vector that spreads malaria and the parasite that causes the disease are sensitive to climate variables especially rainfall and temperature. Research on the impact of climate change on the dynamics of malaria is still ongoing [2–6]. However, most studies tend to consider the effect of temperature alone on the dynamics of malaria, neglecting the impact of incorporating rainfall in the mathematical models of malaria transmission. Understanding the role of temperature and rainfall in malaria transmission is of particular importance in light of climate change as changes can alter vector development rates, shift vector geographical distribution, and alter transmission dynamics. Climate change is widely expected to significantly affect the global spread, intensity, and distribution of malaria. The question is, how is climate change going to affect the gains made thus far in trying to reduce the burden of malaria? That is a question which was once answered by Tanser et al. [7] using spatial models and which we also seek to answer using a mathematical model.

This study seeks to build a mathematical model in conjunction with the use of Geographical Information Systems (GIS) that will enable prediction and mapping of the current and potential future distribution of malaria in Africa as a result of climate change. The study will highlight how combining regional climate models with mathematical models of malaria transmission provides valuable tools for better understanding of future disease scenarios as climatic conditions change. We modify our previous work [4] to incorporate rainfall into the model. We focus on the construction of a realistic, climate-based malaria transmission model that captures the combined effects of both rainfall and temperature on malaria infection dynamics. This approach permits us to gain insight into the effect of climate change on malaria transmission. However, to reduce the complexity of the model genetic variation in vector competences is not explored in the same way as rainfall is not partitioned into runoff and infiltration in this paper.

## 2. Model Description

A deterministic transmission model is developed as a framework for understanding the impact of temperature and rainfall on malaria dynamics. The human population is subdivided into four classes: susceptible (*S*_*H*_(*t*)), exposed or incubating (*E*_*H*_(*t*)), infectious (*I*_*H*_(*t*)), and recovered individuals who become partially immune (*R*_*H*_(*t*)), with *t* accounting for time in days. Individuals are recruited into the susceptible class at a rate *θ* and individuals die naturally at a rate *μ*_*H*_. The rate of infection of a susceptible individual is dependent on the mosquito's biting rate *a*(*T*, *R*) and the proportion of bites by infectious mosquitoes on susceptible humans that produce infection *b*_*H*_. *T* and *R* account for temperature and rainfall dependence, respectively. Once individuals are infected, they do not automatically become infectious as they do not have gametocytes, but they enter the exposed class *E*_*H*_, where parasites in their bodies are still in the asexual stages. Exposed humans then progress at a rate *κ*_*H*_ to the infectious class, in which they now have gametocytes in their bloodstream making them capable of infecting the susceptible anopheles mosquitoes. Individuals recover through treatment at a rate *α*, where a proportion (1 − *p*) recovers with temporary immunity and the compliment *p* recovers with no temporary immunity. Temporarily immune individuals lose immunity at a rate *γ*. Infected individuals who do not seek treatment die from infection at a rate *η*. Both human and mosquito infections take time to develop into an infectious state. Within host parasite dynamics are weather independent, but within vector parasite dynamics as well as the mosquito life cycle are weather dependent.

The mosquito population is divided into the juvenile (*J*_*M*_(*t*)) and adult population, and the adult population is subdivided into three classes: susceptible (*S*_*M*_(*t*)), exposed (*E*_*M*_(*t*)), and infectious (*I*_*M*_(*t*)). The juvenile stages describe the development of the aquatic stages which mature to become susceptible adult mosquitoes at a rate *β*_*M*_. The rate of infecting a susceptible mosquito depends on the mosquitoes' biting rate *a*(*T*, *R*) and the proportion of bites by susceptible mosquitoes on infected humans that produce infection *b*_*M*_. Susceptible mosquitoes that feed on infectious humans will take gametocytes in blood meals, but as they do not have sporozoites in their salivary glands, they enter into the exposed class *E*_*M*_. After fertilisation, sporozoites are produced and migrate to the salivary glands ready to infect any susceptible host; the vector is then considered as infectious and enters class *I*_*M*_. Mosquitoes die at a rate *μ*_*M*_ which is independent of infection status. Infected mosquitoes are not harmed by the infection and never clear their infection and the infective period of the mosquito ends with its death. The following system of differential equations describes the rainfall and temperature dependent malaria transmission model:

(1) SH′t=θ−λHT,RSHt−μHSHt+pαIHt+γRHt,EH′t=λHT,RSHt−κH+μhEHt,IH′t=κHEHt−μH+α+ηIH′t,RH′t=1−pαIHt−γ+μHRHt,JM′t=βJT,RNMt1−JMtK−μJTJMt−βMT,RJMt,SM′t=βMT,RJMt−λMT,RSMt−μMTSMt,EM′t=λMT,RSMt−κMT+μMTEMt,IM′t=κMTEMt−μMTIMt.

Here, *λ*_*H*_ = *a*(*T*, *R*)*b*_*H*_ *I*_*M*_(*t*)/*N*_*M*_(*t*), *λ*_*M*_ = *a*(*T*, *R*)*b*_*M*_ *I*_*H*_(*t*)/*N*_*H*_(*t*), *N*_*H*_(*t*) = (*S*_*H*_ + *E*_*H*_ + *I*_*H*_ + *R*_*H*_)(*t*), and *N*_*M*_(*t*) = (*S*_*M*_ + *E*_*M*_ + *I*_*M*_)(*t*). *X*(*T*, *R*) specifies a function of temperature and rainfall, while *X*(*T*) represents a function of temperature alone.

Predicting the effect of climate change on malaria dynamics requires a framework that specifically incorporates the role of each climate sensitive parameter. The functional forms of temperature and rainfall dependent parameters are presented in Table 1.

### 2.1. Model Analysis

Following van den Driessche and Watmough [14], the treatment induced reproduction number [*ℛ*_*m*_] of the model in (1) is given by

(2) Rm=aT,RbHκHμMTκMT+μMTaT,RbMκMTκH+μHμH+α+η.

In the absence of treatment *α* = 0; then lim_*α*→0_ *ℛ*_*m*_ = *ℛ*_0_, the basic reproduction number. The treatment induced reproduction number defines the average number of new infections a single infected mosquito/individual would produce during its/his (her) entire infectious period where treatment is the only intervention strategy.

## 3. Mapping Transmission Dynamics across Africa

We applied (2) to gridded temperature and precipitation datasets for the baseline climate and future climate to compute *R*_0_ for each pixel in a GIS. The datasets used covered the entire continent of Africa. Data on mean annual temperature and total annual precipitation for the baseline climate (i.e., average values for the period from 1950 to 2000) were downloaded from the WorldClim database as raster grids with a spatial resolution of 30 arc-seconds (approximately 1 km at the equator) [15]. For the future climate, we used temperature and precipitation projections of the HadCM3 and CSIRO Mk3 general circulation models (GCM) based on the A2a emission scenario. Data for the future climate (average values for 2020–2039, hereafter 2040) were downloaded from the Intergovernmental panel on climate change (IPCC) database in raster format at the same spatial resolution of 30 arc-seconds [16]. *R*_0_ was calculated separately for the baseline climate and for each GCM model.

To determine whether falciparum malaria will persist or the disease dies out in the future, we evaluated the following boolean expressions on a pixel basis, respectively: *R*_0_ < 1 for the baseline map and *R*_0_ > 1 for the future map; *R*_0_ > 1 for the baseline map and *R*_0_ < 1 for the future map.

This allowed us to classify an area as becoming malaria endemic or malaria-free. All the maps generated in this study are based on the Albers equal-area conic projection. We clipped the *R*_0_ maps by the raster maps of the digital map of dominant vectors to exclude malaria-free areas such as the Sahara Desert (indicate source here). We used ArcGIS 10.1 for all GIS analysis.

## 4. Results

**Figure 1:**
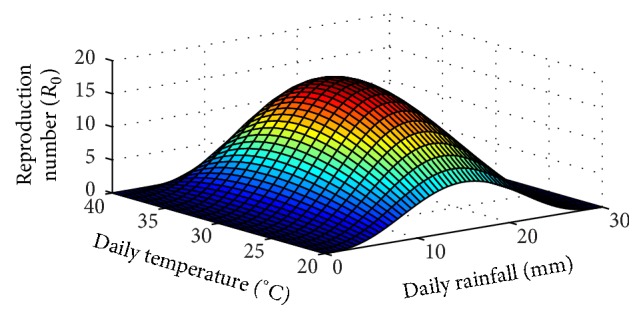
Reproduction number as a function of daily rainfall, *R*, in mm and temperature, *T*, in °C.

In Figure 1, we plot *R*_0_ for falciparum malaria as a function of rainfall and temperature. We observe that the optimum temperature window for falciparum malaria transmission is 30–32°C.

**Figure 2:**
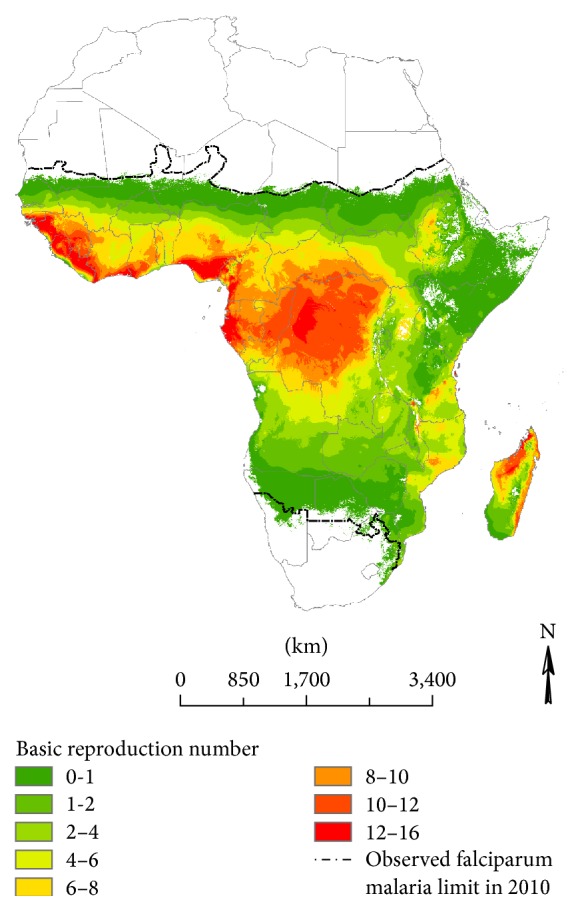
Basic reproduction number for falciparum malaria based on the baseline climate.

Figure 2 illustrates the simulated *R*_0_ for falciparum malaria on the African continent based on baseline climate. We observe distinct geographic patterns in the intensity of falciparum malaria. The transmission intensity is highest in the tropics as well as the coastal areas of East Africa. The subtropics exhibit low levels of transmission intensity. Our simulations fall within the observed spatial distribution of falciparum malaria on the continent described by Gething et al. [17].

**Figure 3:**
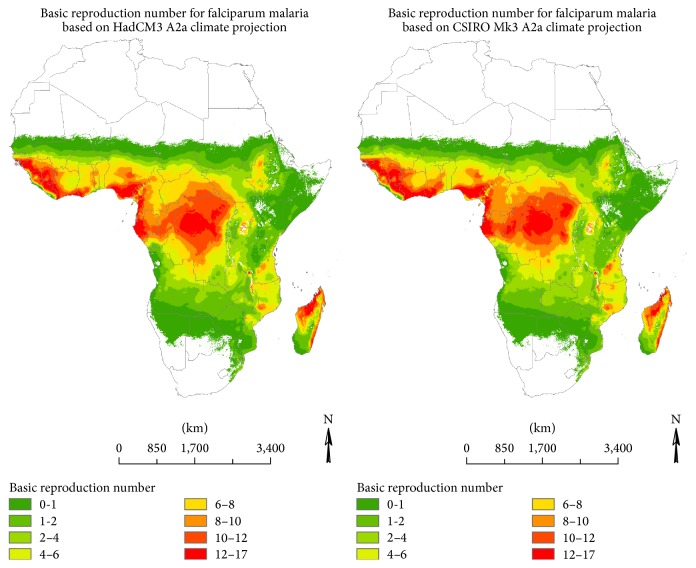
2040 projected basic reproduction number for falciparum malaria.

Figure 3 shows the projected *R*_0_ for falciparum malaria in Africa based on HadCM3 and CSIRO Mk3 A2a climate scenarios for 2040. Compared to the simulations for the baseline climate, we observe increases in *R*_0_ in the tropics, the highland regions, and East Africa as well as along the northern limit of falciparum malaria. By contrast, a decrease in *R*_0_ is projected to occur on the southern fringe of the disease by 2040. These changes are similar for both HadCM3 and CSIRO Mk3 A2a climate projections.

**Figure 4:**
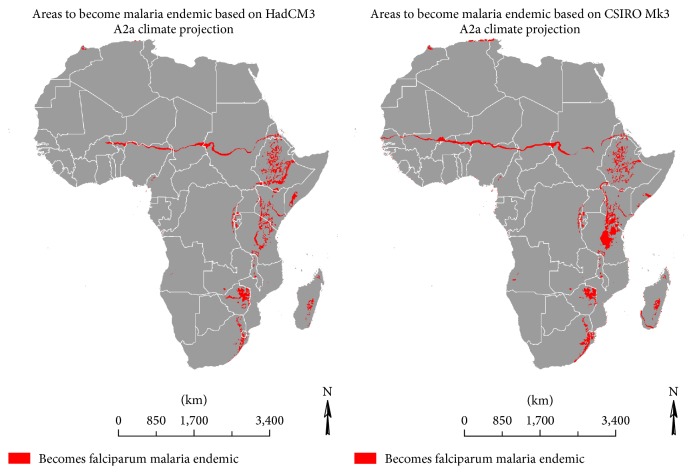
2040 projected malaria endemic areas, previously malaria-free.

In Figure 4 we notice that the increases in *R*_0_ are sufficient to turn most areas in the African highlands into malaria endemic areas by 2040. The northern limit of falciparum malaria is also projected to become an endemic region.

**Figure 5:**
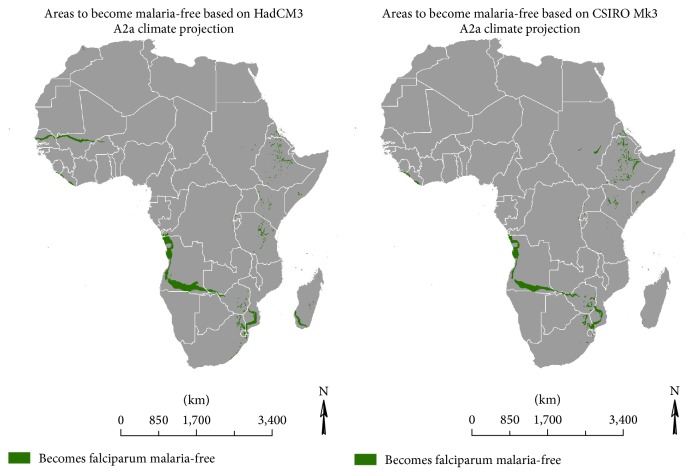
2040 projected malaria-free areas, previously malaria endemic.

In contrast, the decreases in *R*_0_ are sufficient to turn areas that fringe the southern limit of the disease into malaria-free zones. A similar trend is expected for isolated areas in the African highlands as noted in Figure 5.

## 5. Discussion

A model incorporating rainfall and temperature is analysed regarding malaria transmission. Results from the model suggest that the optimum temperature window for peak falciparum malaria transmission is 30–32°C. This is in agreement with other studies [4, 5]. Furthermore, results from model analysis suggest that daily rainfall in the range of 15–17 mm is ideal for the spread of malaria. Perhaps the most interesting but unexpected result is that by 2040 malaria is projected to die out on the southern fringe of the disease in Africa. The fact that the same result was detected using projections from two different GCM models makes this a key result. A drying trend is the likely driving force for this change [18]. This finding has implications for malaria elimination in some regions of Africa. In other words, the result offers hope that the international goal of shrinking the malaria map may be achieved in southern Africa.

Results of this study suggest that due to climate change endemic malaria will become an increasing problem in the African highlands; this seems to be in agreement with other studies [5, 7, 19–22]. A warming trend is the likely factor driving the projected increase in malaria endemicity in the highlands, though socioeconomic factors such as land use change and drug resistance can also be attributed to increases in malaria incidences in highlands too.

The model has the following limitations: (i) it did not consider the role of human migration neither did it consider other climate variables in particular relative humidity as the tropical anopheline mosquitoes prefer humidities above 60% [23]; (ii) it did not consider the role of socioeconomic factors in malaria transmission dynamics but it would be interesting to incorporate these factors to ascertain whether climate change in combination with these factors will amplify malaria transmission in the highlands. Despite these limitations, the model is reasonable enough to be able to give a realistic picture of malaria in the African continent. Thus, results from the study will be useful at various levels of decision making, for example, in setting up an early warning and sustainable strategies for climate change and adaptation for malaria vectors control programmes in Africa. These results can be generalized to other tropical regions outside Africa.

## Figures and Tables

**Table 1:** Parameters of the basic malaria model presented obtained from published literature. (*R*) shows dependence on rainfall and (*T*) represents dependence on temperature.

Description	Symbol	Value	Source
Adult mosquito birth rate	*β*_*M*_(*T*, *R*)	*B*_*E*_ *P*_*E*_(*R*)*P*_*L*_(*R*, *T*)*P*_*P*_(*R*)/(*τ*_*E*_ + *τ*_*L*_(*T*) + *τ*_*P*_)	[8]
Birth rate of juveniles	*β*_*J*_(*T*, *R*)	10*∗β*_*M*_(*T*, *R*)	[9]
Mosquito biting rate	*a*(*T*, *R*)	*β*_*J*_(*T*, *R*)/2.325	[9]
Number of eggs laid per adult oviposition	*B*_*E*_	200	[8]
Daily survival probability of eggs	*P*_*E*_(*R*)	(4*∗*0.9/*R*_*L*_^2^)*R*(*R*_*L*_ − *R*)	[8]
Daily survival probability of larvae	*P*_*L*_(*T*, *R*)	(4*∗*0.25/*R*_*L*_^2^)*R*(*R*_*L*_ − *R*)*∗e* ^−0.00554*T*−0.06737^	[8]
Daily survival probability of pupae	*P*_*P*_(*R*)	(4*∗*0.75/*R*_*L*_^2^)*R*(*R*_*L*_ − *R*)	[8]
Duration of egg development	*τ*_*E*_	1	[8]
Duration of larvae development	*τ*_*L*_(*T*)	1/(0.00554*T* − 0.06737)	[8]
Rainfall beyond which no immature stages survive	*R*_*L*_	50	[8]
Duration of pupae development	*τ*_*P*_	1	[8]
Juvenile mosquito death rate	*μ*_*J*_(*T*)	0.0025*T* ^2^ − 0.094*T* + 1.0257	[9]
Adult mosquito death rate	*μ*_*M*_(*T*)	−ln⁡*ρ*(*T*)	[10]
	*ρ*(*T*)	*e* ^−1/(−0.03*T*^2^+1.31*T*−4.4)^	[10]
Progression rate of mosquitoes	*κ*_*M*_	(*T* − *T*_min_)/*DD*	[11]
Recruitment rate of humans	*θ*	0.028	[12]
Proportion of bites by infectious mosquitoes on susceptible humans that produce infection	*b*_*H*_	0.09	[5]
Proportion of bites by susceptible mosquitoes on infected humans that produce infection	*b*_*M*_	0.04	[5]
Per capita natural death rate for humans	*μ*_*H*_	0.00004	[12]
Progression rate of humans from exposed class	*κ*_*H*_	1/14	[12]
Recovery rate of humans	*α*	0.005	[12]
Per capita disease induced death rate	*η*	0.0004	[12]
Per capita rate of loss of immunity	*γ*	1/(20*∗*365)	[13]
Carrying capacity of larvae	*K*	1000000	[4]
Proportion of humans recovering without temp. immunity	*p*	0.25	[4]

## References

[1] http://www.who.int/malaria/publications/world-malaria-report-2014/en/.

[2] Craig M., Le Sueur D., Snow B. (1999). A climate-based distribution model of malaria transmission in sub-Saharan Africa. *Parasitology Today*.

[3] Martens W. J. M., Jetten T. H., Focks D. A. (1997). Sensitivity of malaria, schistosomiasis and dengue to global warming. *Climatic Change*.

[4] Ngarakana-Gwasira E. T., Bhunu C. P., Mashonjowa E. (2013). Assessing the impact of temperature on malaria transmission dynamics. *Afrika Matematika*.

[5] Parham P. E., Michael E. (2010). Modeling the effects of weather and climate change on malaria transmission. *Environmental Health Perspectives*.

[6] Paaijmans K. P., Cator L. J., Thomas M. B. (2013). Temperature-dependent pre-bloodmeal period and temperature-driven asynchrony between parasite development and mosquito biting rate reduce malaria transmission intensity. *PLoS ONE*.

[7] Tanser F. C., Sharp B., Le Sueur D. (2003). Potential effect of climate change on malaria transmission in Africa. *The Lancet*.

[8] Parham P. E., Michael E. (2010). Modelling climate change and malaria transmission. *Advances in Experimental Medicine and Biology*.

[9] Rubel F., Brugger K., Hantel M. (2008). Explaining Usutu virus dynamics in Austria: model development and calibration. *Preventive Veterinary Medicine*.

[10] Martens W. J. M., Niessen L. W., Rotmans J., Jetten T. H., McMichael A. J. (1995). Potential impact of global climate change on malaria risk. *Environmental Health Perspectives*.

[11] McDonald G. (1957). *The Epidemiology and Control of Malaria*.

[12] Chiyaka C., Tchuenche J. M., Garira W., Dube S. (2008). A mathematical analysis of the effects of control strategies on the transmission dynamics of malaria. *Applied Mathematics and Computation*.

[13] Blayneh K., Cao Y., Kwon H.-D. (2009). Optimal control of vector-borne diseases: treatment and prevention. *Discrete and Continuous Dynamical Systems B*.

[14] van den Driessche P., Watmough J. (2002). Reproduction numbers and sub-threshold endemic equilibria for compartmental models of disease transmission. *Mathematical Biosciences*.

[15] http://www.worldclim.org/.

[16] http://www.ipcc-data.org.

[17] Gething P. W., Patil A. P., Smith D. L. (2011). A new world malaria map: *Plasmodium falciparum* endemicity in 2010. *Malaria Journal*.

[18] Ermert V., Fink A. H., Morse A. P., Paeth H. (2012). The impact of regional climate change on malaria risk due to greenhouse forcing and land-use changes in tropical Africa. *Environmental Health Perspectives*.

[19] Hay S. I., Cox J., Rogers D. J. (2002). Climate change and the resurgence of malaria in the East African highlands. *Nature*.

[20] Lindsay S. W., Martens W. J. M. (1998). Malaria in the African highlands: past, present and future. *Bulletin of the World Health Organization*.

[21] Siraj A. S., Santos-Vega M., Bouma M. J., Yadeta D., Ruiz Carrascal D., Pascual M. (2014). Altitudinal changes in malaria incidence in highlands of Ethiopia and Colombia. *Science*.

[22] Thomas C. J., Davies G., Dunn C. E. (2004). Mixed picture for changes in stable malaria distribution with future climate in Africa. *Trends in Parasitology*.

[23] Martens P., Thomas C., Takken W., Martens P., Bogers R. (2005). Climate change and malaria risk: complexity and scaling. *Environmental Change and Malaria Risk: Global and Local Implications*.

